# AI in Psychiatric Education and Training From 2016 to 2024: Scoping Review of Trends

**DOI:** 10.2196/81517

**Published:** 2025-12-31

**Authors:** Michael James Weightman, Anna Chur-Hansen, Scott Richard Clark

**Affiliations:** 1 Discipline of Psychiatry, Faculty of Health and Medical Sciences, The University of Adelaide, Level 6, Adelaide Health and Medical Sciences Building, Corner of North Terrace and George Street, Adelaide, SA, 5000, Australia, +61 883138163; 2 Central Adelaide Local Health Network, Adelaide, SA, Australia; 3 School of Psychology, Faculty of Health and Medical Sciences, The University of Adelaide, Adelaide, SA, Australia

**Keywords:** artificial intelligence, AI, AI literacy, ChatGPT, psychiatry, medical education, psychiatry education, academic integrity

## Abstract

**Background:**

Artificial intelligence (AI) is rapidly changing both clinical psychiatry and the education of medical professionals. However, little is currently known about how AI is being discussed in the education and training of psychiatry for medical students and doctors around the world.

**Objective:**

This paper aims to provide a snapshot of the available data on this subject as of 2024. A deliberately broad definition of AI was adopted to capture the widest range of relevant literature and applications, including machine learning, natural language processing, and generative AI tools.

**Methods:**

A scoping review was conducted using both peer-reviewed publications from PubMed, Embase, PsycINFO, and Scopus databases, and gray literature sources. The criterion for inclusion was a description of how AI could be applied to education or training in psychiatry.

**Results:**

A total of 26 records published between 2016 and 2024 were included. The key themes identified were (1) the imperative for an AI curriculum for students or doctors training in psychiatry, (2) uses of AI to develop educational resources, (3) uses of AI to develop clinical skills, (4) uses of AI for assessments, (5) academic integrity or ethical considerations surrounding the use of AI, and (6) tensions relating to competing priorities and directions.

**Conclusions:**

Although a nascent field, it is clear that AI will increasingly impact assessment, clinical skills training, and the development of teaching resources in psychiatry. Training curricula will need to reflect the new knowledge and skills required for future clinical practice. Educators will need to be mindful of academic integrity risks and to emphasize development of critical thinking skills. Attitudes of psychiatrists toward the rise of AI in training remain underexplored.

## Introduction

Artificial intelligence (AI), which originated from mid-20th century mathematics and computer science research [1], has experienced an upsurge of interest since the release of ChatGPT in 2022 [2]. The United Nations Educational, Scientific and Cultural Organization defines AI as “machines capable of imitating certain functionalities of human intelligence, including such features as perception, learning, reasoning, problem solving, language interaction, and even producing creative work” [3]. AI can be further delineated into classical AI (rule-based) and newer learning-based AI technologies of increasingly sophisticated function (Table 1). Although tools such as ChatGPT have captured much of the increased interest in this field, generative AI represents only a fraction of available AI technology.

**Table 1.** Explanation of major types of artificial intelligence technology (paraphrased from Miao et al [3]).

Type of AI^a^	Explanation
Rule-based AI	
Classical AI	Rule-based computing systems that use preprogrammed “if-then” rules and conditional logic to perform tasks.
Learning-based AI	
Machine learning	Algorithmic models built from analysis of massive banks of data that are trained to identify patterns and predict future values, without having been preprogrammed.
Artificial neural networks	A subset of machine learning composed of multiple interconnected layers of artificial neurons, which “learn” by adjusting the weights of connections between layers during training. This learning process optimizes the model’s ability to identify patterns and make predictions.
Deep learning	Uses a greater number of layers in artificial neural networks to enable performance of more advanced tasks.

a AI: artificial intelligence.

The impact of AI is growing rapidly and is far-reaching [4,5]. AI is expected to markedly change the clinical practice of psychiatry, through AI-automated diagnostic procedures or precision psychiatry [6,7] and development of chatbot psychotherapy [8,9]. Similarly, medical educators are discussing the substantial pedagogical impacts arising from AI [2,10].

To date, there has been limited exploration of how AI has affected teaching and learning specific to psychiatry. A recent scoping review identified only 5 relevant papers [11]; however, this review exclusively focused on generative AI, which risks neglecting the myriad other types of AI tools. A broader search of the literature pertaining to AI in psychiatric pedagogy would be valuable, as it may be more likely to reveal potential differences between psychiatry and other areas of medical education. Although a biopsychosocial discipline [12], psychiatry suffers from conflicting explanatory models and ontological disagreements over the mind-brain axis [13]. Due to its focus on the individual human experience and the subjectivity inherent in it [14], some attributes of psychiatry may be considered less replicable by machines. For example, psychiatry has traditionally relied on descriptive phenomenology over organic pathophysiology [14] and emphasizes interpersonal connection and empathy clinically [15]. In this context, it is anticipated that the impact of AI on the education of psychiatrists may differ in some respects from education in other medical disciplines.

This study aims to summarize current knowledge on the use of AI within psychiatric pedagogy using a deliberately broad definition of learning-based AI technologies. Due to the nascent nature of the field and expected substantial heterogeneity within the available literature, a scoping review of the relevant literature was performed. We sought to answer the research question, “How is AI being discussed in the education and training of psychiatry for medical students and doctors around the world in 2024?”

## Methods

### Study Design

This review adhered to the Arksey and O’Malley methodological framework for scoping reviews, following the stages of (1) identifying the research question, (2) identifying relevant studies, (3) study selection, (4) charting the data, and (5) collating, summarizing, and reporting the results [16]. The review protocol was predesigned but not formally registered. The PAGER (Patterns, Advances, Gaps, Evidence for practice and Research recommendations) framework was used to improve the quality of reporting [17], and checklist items from the PRISMA (Preferred Reporting Items for Systematic Reviews and Meta-Analyses) were adopted where applicable to enhance rigor [18,19].

### Eligibility Criteria

Records were sought from both peer-reviewed literature and gray literature sources. A record was deemed eligible for inclusion if it described an application of learning-based AI and if the listed application was relevant to education or training in psychiatry. Records were excluded if they were not available as a full text, were conference abstracts, were written in a language other than English, or were considered only classical AI.

### Search Strategy

Electronic searches were performed using PubMed, Embase, PsycINFO, and Scopus databases. An expert librarian (VL) assisted with the design of the search strategy and terms. Keyword search terms included “artificial intelligence,” “psychiatry,” and “training,” as well as common interchangeable terms. For example, “large language model,” “machine learning,” “deep learning,” “chatGPT,” and “chatbot” were used in addition to “artificial intelligence,” and “education,” “teaching,” “learning,” and “assessment” were used in addition to “training.” The Boolean operator “OR” and truncated search terms were included where possible to maximize results. In addition, subject headings relating to “artificial intelligence,” “psychiatry,” and “medical education” were tailored for each database. No limits were placed on the searches. Refer to Multimedia Appendix 1 for the precise search syntax used for each of the 4 included databases.

The search was conducted on May 8, 2024, and pertinent records were retrieved from each of the included databases. Titles and abstracts were screened for relevance, with full texts scrutinized for confirmation of eligibility. The reference list of each included record was scoured to identify any additional eligible records.

Relevant gray literature was identified via 2 separate strategies. First, an electronic search was conducted using Google search engine on June 8, 2024, to identify eligible internet records meeting the inclusion criteria. Second, professional organizations responsible for psychiatry training across major English-speaking jurisdictions globally were contacted to provide copies of any relevant documents or reports. Institutions were included if their psychiatry training program was predominantly conducted in English and if contact details were published on their institutional website. Nine organizations from Europe, North America, Asia, Africa, and Australasia were identified. Initial correspondence was sent on June 8, 2024, with follow-up a month later if no response had been received. Refer to Multimedia Appendix 1 for additional details of the gray literature search.

### Data Collection, Analysis, and Synthesis

All records meeting inclusion criteria were read in full by MJW and data relevant to the research question were extracted into a Microsoft Word document, along with relevant demographic details. ACH and SRC cross-checked the extracted content from the included records to ensure accuracy. Data categories of interest included author name, year of publication, country of research group, type of publication (ie, journal, book, or report), population of interest, application of AI technology, methodology, key findings, and suggestions for future research.

 Extracted data were assessed against the research question to determine patterns, advances, gaps, evidence for practice, and research recommendations as per the PAGER framework [17]. These data were then synthesized and presented as core themes.

## Results

### Overview

**Figure 1.**
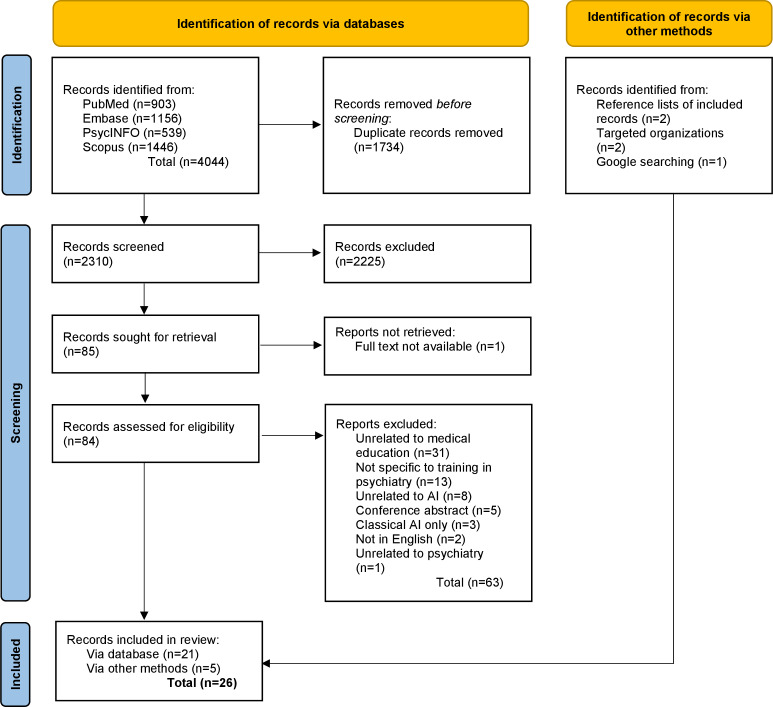
PRISMA (Preferred Reporting Items for Systematic Reviews and Meta-Analyses) flowchart (adapted from Page et al [18]). AI: artificial intelligence.

A total of 26 records were included, with the screening and selection process depicted in Figure 1. The primary reason for exclusion was a lack of educational applications for the AI intervention. From the 9 organizations approached for gray literature, 2 relevant documents were received, 3 organizations responded that they did not yet have a formal policy document relating to AI, and another provided a document pertaining to AI that explored clinical applications rather than training considerations.

A detailed summary of all included records is shown in Multimedia Appendix 2. In brief, dates of publication ranged from 2016 to 2024, although this was skewed to more recent years with 16 of the 26 records (61.5%) published in either 2023 or 2024. The majority of records were authored by North American (10/26, 38.5%) or European researchers (8/26, 30.8%), with the remaining from Australia (4/26, 15.4%), Asia (2/26, 7.7%), South America (1/26, 3.8%), and Israel (1/26, 3.8%). Of the peer-reviewed literature (n=24), most records had been published in psychiatric journals (17/24, 70.8%) or books (1/24, 4.2%), although digital health (3/24, 12.5%), medical education (2/24, 8.3%), and criminology (1/24, 4.2%) journals were also represented. The most common type of record was an opinion piece (eg, editorial or perspective paper; 13/26, 50%). Other methodologies included quantitative (6/26, 23.1%), mixed methods (3/26, 11.5%), qualitative (1/26, 3.8%), and other (3/26, 11.5%). The key themes identified across the 26 records are shown in Table 2 using the PAGER framework.

**Table 2.** Summary of themes using the PAGER (Patterns, Advances, Gaps, Evidence for practice and Research recommendations) framework (adapted from Bradbury-Jones et al [17]).

Pattern	Advances	Gaps	Evidence for practice	Research recommendations
The imperative to develop an AI^a^ curriculum for students or doctors training in psychiatry	There is some limited qualitative and survey evidence that psychiatrists desire increased training on AI and believe that it should be a priority topic in training curricula.	Topics on AI literacy and clinical informatics are lacking from the majority of psychiatry curricula. Specific training will be required to instruct psychiatry trainees on how to safely and effectively incorporate AI tools into clinical care.	Educators in psychiatry will need to ensure that AI skills and knowledge are covered within psychiatry training.	To carry out Delphi expert opinion studies and stakeholder focus groups (eg, with trainees or students) to develop key competencies relevant to AI for incorporation into psychiatry curricula.
Uses of AI to develop educational resources	Multiple authors have described how they have used AI to produce case vignettes, build a curriculum, or create resources for learners.	There is a current lack of evidence evaluating the use and rigor of the educational resources being generated through AI.	Educators will need to be mindful of risks associated with AI-generated resources, such as inaccuracy, bias, and reduction in critical thinking.	To carry out surveys and thematic analysis relating to educational resources generated through AI to evaluate their quality and use.
Uses of AI to develop clinical skills	Addition of NLP^b^ to virtual reality psychiatry skills training appears to increase the ecological validity of the task.	Technological limitations constrain increased uptake of AI-enhanced simulated training currently, but such limitations are expected to be overcome with time.	AI-enhanced simulated training can provide benefits relating to increased standardization, safe access for the learner to risky or rare scenarios, and reduced iatrogenic harm to patients.	To carry out more research incorporating NLP into various clinical skills training modalities in psychiatry, leveraging concurrent technological advances.
Uses of AI for assessments	Overall, generative AI (especially ChatGPT) has performed well on MCQ^c^, SAQ,^d^ and clinical scenario assessments in psychiatry (particularly at medical student level in comparison with specialist training level).	Minimal research exists on evaluating the ability of AI tools to generate assessment tasks for learners in psychiatry.	Educators must be aware that generative AI is able to perform competently on many extant assessment types, which may have implications for the effectiveness of current assessments.	To carry out surveys and thematic analysis to validate the quality and use of generating questions for psychiatry assessments (eg, MCQ or OSCE^e^).
Academic integrity or ethical considerations surrounding the use of AI	The Hong Kong Academy of Medicine has developed a comprehensive policy on use of generative AI in written assessments, which may provide a template for other jurisdictions.	Most specialist training organizations in psychiatry do not have formal policies or position statements relating to ethical use of AI within training. There is a current lack of regulation around AI tools with clinical applications.	It is important to build a culture of declaring whether and how AI was used as part of submitted work. Written assessment tasks may need adaptation to include an oral or viva voce component to ensure assessment rigor.	To carry out research and development creating bespoke LLMs^f^ at a local institutional level that ensure absolute confidentiality and privacy of clinical information that may be fed into the model.
Tensions relating to competing priorities and directions	N/A^g^	Uncertainty over how to balance traditional teaching practices with embracing the opportunities of novel AI technology. Attitudes of psychiatrists toward AI are largely unknown.	The interpersonal nature of psychiatry remains an important defining feature, but the nature of the profession will inevitably change in the face of technological advancement.	To carry out qualitative research with psychiatrists, trainees, and medical students to understand their perceptions and attitudes toward the use of AI in the teaching of psychiatry.

a AI: artificial intelligence.

b NLP: natural language processing.

c MCQ: multiple choice question.

d SAQ: short answer question.

e OSCE: objective structured clinical examination.

f LLM: large language model.

g Not applicable.

### Imperative for an AI Curriculum

The most frequent theme (10/26 records, 38.5%) was the need to ensure that learning about AI becomes a key component of the curriculum for medical students and doctors training in psychiatry [20-29]. Both Louie et al [24] and Torous et al [27] argued that psychiatrists of the future will need to be competent in using available AI tools in patient care (eg, diagnosis, monitoring, and clinical decision-making) and that training will need to adapt to accommodate such changes. Gauld et al [20] proposed that new areas of learning might include how to (1) identify clinically relevant AI tools, (2) interpret and understand the outputs, and (3) discuss outputs with patients or multidisciplinary colleagues. Separately, Torous et al [27] developed prototype core competencies for AI in psychiatric training (Table 3) based on the United States’ Accreditation Council for Graduate Medical Education framework. Torous et al [27] further argued that this content would likely sit within the broader discipline of clinical informatics, which they considered an area of deficiency within psychiatry curricula.

Khanna et al [22] observed that, while medical schools and psychiatric training organizations will need to ensure that their graduates are equipped with the general knowledge and skills required to navigate increased expectations of proficiency with AI, psychiatrists do not necessarily require expert-level knowledge. Louie et al [24] also expressed uncertainty about what degree of granularity is reasonable for future psychiatric curricula, as it is unclear how much oversight generalist psychiatrists will be expected to have in terms of understanding and explaining to patients issues around data privacy and potential side effects of use.

**Table 3.** Proposed competencies for artificial intelligence as based on the Accreditation Council for Graduate Medical Education framework (adapted from Torous et al [27]).

ACGME^a^ competencies	Opportunities to incorporate AI^b^
Patient Care and Procedural Skills	Use LLM^c^ chatbots to summarize information on a topic. Use of NLP^d^ to enhance virtual and augmented reality clinical skills training.
Medical Knowledge	Understand how AI tools can optimize or hinder workflows.
Practice-Based Learning and Improvement	Use AI tools to optimize learning and self-care. Educate patients on AI tools.
Interpersonal and Communication Skills	Explain risks and benefits of AI technology to patients, medical students, and multidisciplinary colleagues.
Professionalism	Demonstrate sensitivity to the impact of AI on psychotherapy, clinical practice, psychiatrist-patient relations, and work-life balance.
Systems-Based Practice	Use AI systems to prevent adverse events.

a ACGME: Accreditation Council for Graduate Medical Education.

b AI: artificial intelligence.

c LLM: large language model.

d NLP: natural language processing.

 Gauld et al [20] posited that the integration of AI into psychiatric curricula must be based on a sound epistemological framework that emphasizes clinical reasoning and philosophical or ethical principles. This would ensure that students can critically evaluate the output of AI and recognize that AI relies on prediction rather than inference. Similarly, Lemon [30] argued that helping learners develop critical thinking skills is a fundamental task of an educator, particularly in social psychiatry where historical, political, and social contexts are an essential “lens” through which information (including AI-generated content) must be interpreted. Starke et al [26] also emphasized how a proper understanding of the historical context of psychiatry, especially the limitations of psychiatric nosology, is invaluable in engendering a more critical perspective of AI output in trainee psychiatrists.

Louie et al [24] predicted that psychiatrists of the future will require training on how to integrate new data sources into clinical care. They argued that, currently, psychiatrists rely on cross-sectional assessments of history and mental state; however, access to longitudinal, real-world biometric data through smart devices with subsequent use of machine learning (ML) analysis to reveal patterns could offer a rich additional source of clinically relevant information for psychiatrists to use diagnostically, prognostically, and for monitoring recovery. They observed that the meaning and significance of this additional information will need oversight and interpretation by suitably trained psychiatrists, which will necessitate psychiatry trainees to learn about these developments, the advantages and risks, and how to use them to their patients’ benefit.

Gratzer and Goldbloom [21] argued that, given psychotherapy skills are a core competency of many psychiatry training programs, psychiatry trainees must also become familiar with new chatbot therapy options. Chatbots use ML techniques, such as natural language processing (NLP), to mimic human delivery of manualized psychotherapy. Gratzer and Goldbloom advocated that trainees should gain first-hand experience of using these chatbots, become sufficiently knowledgeable to answer patient questions, be able to prescribe a suitable option for their patient, and appropriately oversee their patient’s use of this therapy. They also argued that psychiatry trainees must be aware of important ethical considerations, such as the privacy of patient information, the credibility of individual apps, and absence of the boundaries typical within traditional therapy.

Starke et al [26] acknowledged that psychiatric curricula will be required to cover progressively more topics as time goes on, so additional AI content risks overstretching capacity. Conversely, Reznick et al [25] noted that AI may automate or replace certain tasks currently performed by doctors, and redesigned curricula must thus equip doctors with transferable skills that facilitate movement between roles as necessitated by technological advancements. They anticipated increased interdisciplinary collaboration, including with nontraditional partners such as engineers, data scientists, and informaticians.

Most data related to this theme were in the form of expert opinion, although some qualitative and survey evidence was available regarding the attitudes of psychiatrists and other health professionals. Zhang et al [29] conducted qualitative interviews with 20 Ontarian mental health professionals, including 3 psychiatrists. They found that respondents believed that increased training and knowledge of AI would reduce negative perceptions or fears about its uptake. Some respondents worried that their lack of mathematical or informatics knowledge would restrict their ability to learn about AI. Respondents also strongly supported the development of a competency-based AI curriculum specific for mental health professionals and ongoing mentoring to address the knowledge gap. Reznick et al [25] surveyed 4224 Canadian doctors, 8.5% of whom were psychiatrists. They found that 22.5% of surveyed specialists considered that AI education should be a priority topic in residency. This corresponded with 56.9% of residents who indicated that they had received no preparation for AI during their training. Residents selected “technical training related to your specialty” as their highest priority for AI content in their training curriculum. Overall, the surveyed population was positive about the prospects of AI; 49.2% of specialists and 48.7% of residents thought that AI “may disrupt the current workflow in a positive manner,” while 23% and 28%, respectively, stated that it “definitely would have a positive effect.”

### Use of AI for Developing Educational Resources

Five records explored ways in which AI might efficiently produce resources for teaching psychiatry [30-34]. Examples included generating clinical scenarios for teaching purposes, using as a live resource in group tutorials, creating a summary of a topic or concept to provide to students, or generating a curriculum.

Large language models (LLMs), such as ChatGPT, have elicited interest in their educational potential. When Smith et al [33] asked ChatGPT-3.5 to brainstorm ways it could aid teaching in social psychiatry, it identified 6 potential applications (Textbox 1). Several authors have described using an LLM like a teaching assistant by getting it to quickly generate content to support an educator-led tutorial [30,32-34]. For example, Smith et al [33] used ChatGPT-3.5 to generate a plausible case vignette suitable for teaching in social psychiatry. Moreover, LLMs may allow for psychiatry teaching resources in English to be rapidly translated into other languages [34] or the generation of new resources in different languages [33]. Torales and O’Higgins [34] asserted that this may improve current deficits in teaching resources and research findings in languages other than English.

Textbox 1. **Suggestions from ChatGPT about how it could support teaching in psychiatry (adapted from Smith et al [33]).**

Ways that ChatGPT can support teaching in psychiatry:

- By providing up-to-date information and resources
- By facilitating discussions and debates
- By answering questions
- By providing case studies
- By facilitating collaboration and teamwork
- By facilitating self-directed learning

Amos et al [31] explored using ML to generate a psychiatric curriculum. They analyzed self-organizing maps using *k*-means clustering to identify the key themes contained in reference lists from a core psychiatric textbook. They argued that building a curriculum based on data-driven topic selection, rather than expert opinion, may minimize bias and ensure currency of included topics.

Multiple records raised concerns about potential risks or biases that may result from the uptake of AI in psychiatric training. Four records identified that LLMs may be less adept in highly specialized fields, such as social psychiatry [28,30,33,35]. Smith et al [33] raised concern that LLMs may be more likely to provide content aligned with the prevailing paradigm in a particular discipline, such as an overemphasis on biomedical perspectives in psychiatry. Vasilchenko and Chumakov [35] observed that it is unclear how adept extant AI tools are at understanding abstract concepts, interpreting nuance in language, or awareness of group dynamics—all of which they considered to be essential skills for a psychiatrist.

Other potential risks, as identified in the records, include undetected bias within the generated content, inaccurate or “hallucinated” content, poor replicability or reproducibility of generated replies, and the potential for substantially different responses to be obtained from minor alterations in prompt [30,32,33]. Smith et al [33] noted that there is usually a cutoff point for the data used to train an LLM—at the time of writing, 2021 for ChatGPT-3.5—so the most contemporary data are not factored into results. Two records emphasized the importance of human oversight of the process and careful review of any educational content generated [32,34]. Others raised concerns about the lack of explainability regarding how ML algorithms generate output [20,31,33]. Amos et al [31] argue that medical educators are more likely to use ML in their work if they understand the process by which the results were generated.

The consensus from the included records was that use of AI should complement the role of educators, rather than replace them. Lemon [30] described the important ongoing role for educators to ensure that learners are practicing critical thinking skills, as well as to correct for obvious errors and biases in AI content. Smith et al [33] suggested that materials generated from AI can be best used as a “starting point” from which the educator can add nuance, context, and their own knowledge to develop a more “inclusive and comprehensive” result.

Three records recognized that learners may also use AI to develop bespoke educational resources as part of self-directed learning [30,33,35]. These records provided examples of how learners could use LLMs to rapidly summarize all available data on a particular topic or generate a short précis of a specific text. This allows for tailored learning that is less available in larger group-based teaching contexts. However, Vasilchenko and Chumakov [35] noted some potential hazards associated with unsupervised self-guided learning, as it is dependent on the motivation of the student, and the lack of educator oversight risks students relying on biased or inaccurate information.

### Use of AI for Clinical Skills Development

A further theme covered how AI-enhanced technology might assist students and doctors-in-training to learn and hone clinical skills in psychiatry. For example, virtual patients (VPs) enhanced with NLP can simulate clinical interactions with increased fidelity to a real patient [36,37]. Use of NLP allows live generation of authentic responses to the student interviewer, which contrasts to standard VPs that are preprogrammed with only a limited array of potential responses. NLP can also be used to assist training in history taking without an avatar: Espejo [32] describes using ChatGPT in small group teaching to generate real-time responses to student questions from the perspective of a patient with depression.

Beyond history content, a French group incorporated deep learning techniques to analyze students’ facial expressions during a VP-based psychiatric interview task, allowing educators to measure students’ nonverbal empathy skills [38]. While the study showed technological limitations with only 60% of measurements usable, further development of this technology may allow provision of individualized feedback on empathy to students. In contrast, Foster et al [37] incorporated human-rated empathy feedback into an AI-enhanced VP patient assessment task to assist students’ learning.

Barbe et al [36] argued that incorporating NLP to VP platforms can enhance the ecological validity of training through improved realism and standardization of simulated conversational elements. Foster et al [37] asserted that this benefit extends to text-based models. For models with an imagery component, the studies included in the present review use either computer-generated avatars—which are not yet photorealistic [36,38]—or static images [37]. Nevertheless, Foster et al [37] argued that VPs afford certain benefits over standardized- or live-patient interactions; standardized patients can be expensive and limited in availability, while live patients can lack standardization or breadth in the clinical scenarios encountered, and there is potential for iatrogenic harm to the patient. Barbe et al [36] observed that VPs may be particularly suited to certain contexts; for example, forensic psychiatry requires learners to gain skills assessing patients at high risk of violence, which may be difficult or inappropriate to facilitate using real patients. As a counterpoint, authors with a social psychiatry perspective have emphasized the crucial importance of human connection, arguing that interactions with real patients will remain essential for developing communication skills in psychiatry [28,35].

Looking to the future, Schildkrout [39] anticipated that the rapid development of AI technology may soon allow for recorded interactions between a patient and a psychiatrist to be sufficiently deidentified such that the image or video could be widely used as an educational resource. Theoretically, anonymized videos could remove all identifying data while accurately preserving the relevant phenomenological features of the patient’s presentation, including for rare cases that a trainee may not otherwise encounter. Schildkrout appropriately identified important ethical issues that would need to be addressed, such as informed consent procedures, complying with regulatory requirements, and ensuring absolute confidence in the technology’s capability to preserve confidentiality.

### Use of AI for Assessments

Four included records investigated how LLMs performed on extant assessments of learning in psychiatry [40-43]. This has important educational implications, including potentially reduced effectiveness of current assessment modalities.

Two studies investigated the performance of LLMs on answering multiple choice questions (MCQs) [41,42]. At the medical student level, Herrmann-Werner et al [41] demonstrated that ChatGPT-4 achieved high accuracy on German language MCQs by answering more than 90% of questions correctly and passing 16 out of 16 individual examinations. At the specialist trainee level, Li et al [42] found that ChatGPT-4 achieved a passing grade (≥60%) on 3 Mandarin MCQ examinations (60%‐69% across the 3 examinations) but Bard (36%‐42%) and Llama-2 (24%‐34%) did not. The authors noted that all 3 LLMs struggled on more complex MCQ types—such as reverse questions or converting a list of options into a single-choice answer—and that performance on these questions improved when additional prompts were entered into the model. In addition, Bard’s performance in this study was significantly stymied by its refusal to answer questions related to specific taboo subjects, including suicide and domestic violence. Li et al [42] found that ChatGPT was significantly superior to Bard and Llama-2 on questions categorized as “pathophysiology and epidemiology” or “psychopharmacology and other therapies”. The authors speculated that ChatGPT-4’s superior performance may be because it is trained on 1.7 trillion parameters, compared with 147 billion and 65 billion for Bard and Llama-2, respectively. The authors further hypothesized that LLM performance could be improved by pretraining them on specific psychiatric data. Taken together, these 2 studies on MCQs suggest that LLMs may be more accurate at more basic questions aimed at medical students but not yet as strong at answering the more difficult questions set at the specialist level.

Herrmann-Werner et al [41] extended their MCQ performance analysis by instructing ChatGPT to provide reasoning for each answer selected; incorrect answers were then analyzed against Bloom’s taxonomy of educational goals to determine the level at which errors arose. They found that the lower-order cognitive tasks of “remember” (eg, ignoring specific facts) and “understand” (eg, misunderstanding how different facts interact together) were the levels at which ChatGPT most frequently encountered errors, while a smaller proportion of errors related to “apply.” The authors acknowledged that applying a cognitive paradigm to an LLM’s predictive algorithm is an anthropomorphism but contended that this approach may help classify and demonstrate the types of errors that the model can produce.

Two studies investigated LLM performance on clinical scenario questions [40,42]. D’Souza et al [40] sought to validate the performance of ChatGPT-3.5 on answering 100 psychiatric case vignettes, most of which related to management, diagnosis, or differential diagnosis. In general, ChatGPT performed well: 61% of its responses were assessed as “highly acceptable” by covering all aspects of the standard answer key and 31% were assessed as “moderately acceptable” by covering “most of the aspects.” The remaining 8% covered ”only a few of the aspects,” while 0% failed to cover any. Qualitatively, the authors noted ChatGPT’s responses to be “brief” and “relevant.” Li et al [42] compared the diagnostic performance of 24 experienced psychiatrists against the LLMs of ChatGPT-4, Bard, and Llama-2 on a complex clinical scenario. Using Bayesian analysis, they predicted that psychiatrists would outscore all LLMs with at least a 99.2% probability on these scenarios (although ChatGPT-4’s overall performance approached that of the psychiatrist group). However, no qualitative analysis of differences between the psychiatrist and the LLM groups’ answers was conducted in this study.

Luykx et al [43] assessed the ability of ChatGPT (version unspecified) to answer open-ended short answer questions about core psychiatric knowledge. The answers generated by ChatGPT were rated highly for accuracy (8.0/10, 80%), completeness (7.6/10, 76%), and nuance (8.1/10, 81%). As a follow-up analysis, Luykx et al also assessed the performance of 38 psychiatrists and psychiatrists-in-training who were randomized to use either ChatGPT exclusively as their source or any other nonchatbot source of their choosing when answering short answer questions. Participants who used ChatGPT scored significantly better than those who used non-AI sources (*P*=.0016) and were also 19% quicker at answering the questions (nonsignificant, although the study may be underpowered). Interestingly, ChatGPT users were less accurate on psychopharmacology questions; Luykx et al speculated that this could be due to a relative lack of pharmacology sources in ChatGPT’s training data.

A further application for AI in the assessment space is in designing assessments of learning in psychiatry. Hudon et al [44] used ChatGPT-3.5 to design psychiatric script concordance test (SCT) questions for medical students. They found that SCT questions generated by ChatGPT were rated equivalently to those generated by clinical experts for both the scenario and question components. Independent assessors were not able to reliably determine authorship between human- and ChatGPT-generated questions. They also found that both groups of questions received similar qualitative feedback in that they were appropriate diagnostically but relatively simplistic in content. Hudon et al argued that, because SCT questions are time-consuming to produce, LLMs potentially may make this process more efficient.

### Academic Integrity and Ethical Considerations

The Hong Kong Academy of Medicine (HKAM) is the only national association responsible for psychiatric training that appears to have a policy on AI use for its trainees at the time of data collection for this review. The HKAM policy covers written assignments in specialist training [45]. It emphasizes the responsibility of trainees to ensure originality of their work, to critically appraise or validate any output of AI tools that may be used in the preparation of their work, and to fully disclose any such use of generative AI. It outlines disciplinary steps if trainees do not adhere to these principles. The policy also specifies that the HKAM intends to develop alternate modes of assessment to minimize the potential for AI to be misused; this includes use of oral presentations or viva voce assessments as an adjunct to written assignments. Multiple other national training organizations indicated that they were currently developing such a policy or intending to do so (personal correspondence with MJW).

Some authors raised ethical concerns relating to data privacy and storage [24,33]. Smith et al [33] advocated for medical educational institutions at the local level to develop regulations and policies to guide the use of sensitive data with LLMs. Without such safeguards, they argued that there are significant privacy and security risks associated with entering data from real patients into LLM chatbots, such as when developing case vignettes or problem-based learning.

Louie et al [24] noted that AI tools—like chatbot therapists—are generally not formally classified as medical devices by regulatory bodies, such as the United States’ Food and Drug Administration. Louie et al argued that there is insufficient oversight around the safety or claims of benefit for these interventions, which has implications for training, given that psychiatrists maintain a responsibility for assisting their patients to make informed choices about the mental health products they use.

Espejo [32] identified that increased uptake of AI in psychiatric education has the potential to impair student learning. For example, she argued that reliance on AI-generated information can be reductionistic through negatively impacting creativity in thinking and discouraging use of primary sources. Lemon [30] observed that educators cannot control how learners use technology outside the classroom, including the possibility that students or trainees will inappropriately rely on AI in clinical contexts even if instructed not to do so. He hypothesized that incorporating AI tools into psychiatric education could inadvertently normalize their use within clinical contexts, even if this is not their intended application.

Four records noted how the existence of free-to-use AI tools may provide potential equity benefits, as individuals in low-resource settings with otherwise limited access to academic literature now have use of a powerful research assistant [24,30,33,43]. However, as Smith et al [33] pointed out, this benefit is already undermined by companies requiring subscriptions for premium models. Louie et al [24] also noted that those with better internet service provision, more sophisticated software or hardware, and greater digital literacy will hold an advantage.

### Tensions

As Louie et al [24] describe, spending time with patients to talk with them and observe their mental state has always been fundamental to clinical training in psychiatry. While some authors in the present review emphasize the importance of maintaining primacy of this human element of the profession [28], others observe that technological advancements may allow these core skills to be supplemented by new AI capabilities [24]. Nevertheless, both author groups agree that a challenge for educators of the future is to strike an appropriate balance between maintaining traditional teaching practices and choosing which advancements to incorporate into training or how best to do so. This tension was also seen in the qualitative analysis of Zhang et al [29], with some mental health professionals in this study expressing concern that use of AI might negatively impact the therapeutic relationship with their patient, while other respondents considered that education on how to incorporate AI into their practice would be empowering and beneficial.

Kim et al [23] referred to the common belief that psychiatry is more likely to be “immune from technological invasion” than other specialties due to its interpersonal nature and the necessity for rapport. They further speculated that AI’s capacity to develop empathy could become the “Turing test” within psychiatry; in other words, an AI tool’s empathic ability may become the measure by which its “humanness” can be assessed against a psychiatrist. Other authors explored a similar tension in that different skills will be required for navigating technology compared with the skills required for human interaction [32,37].

Authors such as Espejo [32] also discussed the tension that exists between concerns about AI impairing learning compared with the exciting potential for it to enhance the way psychiatry is taught. Instead of resisting encroachment of AI into the teaching space, Espejo encouraged finding ways to use AI for enhancing teaching, for example, using material generated by ChatGPT to start a group discussion or stimulate debate. On this point, Kim et al [23] drew an analogy with how the onset of personal computers heralded a significant shift from paper-based to electronic records; similarly, they argued that educators must adapt to technological advances lest they become outdated.

Louie et al [24] predicted that a proportion of psychiatrists will be reluctant to embrace AI in both clinical practice and education. Although the broader attitudes of psychiatrists to the wider adoption of this nascent technology are understudied, one qualitative analysis of mental health professionals’ attitudes toward AI identified the theme of fear within some participants [29]. Nevertheless, Torales and O’Higgins [34] pointed out that AI is clearly now firmly established and will continue to develop, regardless of the concerns raised by some sectors of the profession. Finally, Smith et al [33] predicted that successive versions of current AI tools and new AI technology that will be developed in the future are likely to be refined and improved so that the current issues with accuracy, bias, and reproducibility will be minimized and potentially eliminated.

## Discussion

### Principal Results

The included literature displayed considerable breadth, but at times limited depth, with respect to the role of AI in psychiatric education for medical students and specialist trainees. Breadth was demonstrated by the wide variety of themes identified and the diverse range of applications described. However, the data were limited in that many of the included records were proof-of-concept studies or opinion pieces. This reflects the infancy of the field, given that more than three-fifths of the included records were published in 2023 or later. It is anticipated that research findings pertaining to AI are liable to change or become quickly outdated, as technology rapidly advances or as current AI tools are updated to new versions. This will pose an ongoing challenge to maintaining currency of knowledge.

It is notable that much of the available data included in this review are speculative or opinion based. While this is helpful for identifying potential research questions, there is a clear need for methodologically rigorous qualitative research to better understand the wider attitudes of psychiatrists and students or trainees toward AI. The evidence available suggests that there is a strong desire for more training in this area and that those less knowledgeable about AI may have greater cautiousness or even fear about its use [25,29].

Similarly, the included quantitative studies are largely proof-of-concept and require replication or present opportunities for more detailed analysis. For example, one study did not go beyond comparing simple accuracy rates between an LLM and human comparator group when answering clinical scenario questions [42], while another was drawn to the clinical implications of AI being able to pass psychiatric examinations rather than the potential educational impact [40]. Future studies have the opportunity to consider the qualitative differences between LLM and human answers on assessment tasks in psychiatry. For example, the exploration of ChatGPT’s output through a Bloom’s taxonomy lens by Herrmann-Werner et al [41] is a helpful illustration of how the quality of AI responses can be further interrogated. Further exploration in this area may also help achieve improved explainability for AI outputs, which is well acknowledged to be an issue with many ML models [46].

The rapid pace of technological development presents substantial challenges for conducting rigorous empirical research using AI. These challenges include the rate of updates to AI models greatly exceeding the traditional timeline from conception to eventual publication of a research project, as well as issues with reproducibility and explainability of findings [47]. A practical step toward addressing some of these issues may be wider adoption of standardized reporting for AI-based research in psychiatry education, such as using the framework proposed by Gordon et al [48], in order to maximize the relevance, interpretability, and reproducibility of future empirical studies.

Many findings from this review can be considered according to the impact on the *educator* (ie, academic psychiatrists or clinician-educators) or the *learner* (ie, medical students or doctors specializing in psychiatry). For educators, AI can be a useful adjunct to produce resources (such as case vignettes, examination questions, or curricula) or enhance training techniques (such as adding NLP to virtual interview practice). For students, AI can become a personal tutor to help facilitate self-guided learning and potentially provide sophisticated feedback, in real time, on clinical and academic performance.

In contrast, a minority of included records considered the potential impact of AI on *psychiatry* itself. AI has the potential to be a phenomenon of influence on human behavior directly in both predictable and unpredictable ways. For example, patients will be increasingly likely to have consulted AI prior to seeking psychiatric help, similar to the earlier emergence of “Dr Google” [49]. It is also foreseeable that entirely new disorders may arise from problematic use of AI, with “AI psychosis” [50] and harmful use of AI companions [51] being just 2 emerging examples. Some authors included in this review identified the need for trainee psychiatrists to be taught how to counsel patients about the appropriate use of AI interventions or under what circumstances an AI therapy bot may be safely prescribed. These skills and knowledge will need to be sufficiently transferable to apply in future scenarios that cannot currently be anticipated. Furthermore, these new skills are likely to be unfamiliar and, perhaps, uncomfortable to experienced educators, which may necessitate specific formal education beyond training programs (such as inclusion into continuing professional development for fully qualified psychiatrists).

It is plausible that increased uptake of AI in psychiatric education might further define the role of educators. As Masters [10] observed, the task of an educator has moved well beyond simply transferring knowledge to the learner. As identified by authors in this review, educators may achieve increased efficiency through using AI to perform time-consuming tasks. This may reflect Moravec’s paradox: tasks that humans find difficult or time-consuming (eg, complex data computation) are easy for AI to perform, while tasks that humans are skilled at (eg, interpersonal skills) are difficult for AI to replicate [52]. Multiple authors in this review identified a crucial human role for monitoring AI outputs and adjusting or improving them as necessary. AI is not yet at the stage where it can be used unsupervised, especially in the context of the well-documented technical and ethical concerns relating to AI’s biases, accuracy, and reproducibility. These concerns are not unique to psychiatry and have been commented on extensively in the wider literature [47,53,54]. While not flagged by the authors of records included in this review, the student-teacher relationship is central to learning to be a medical practitioner [55,56], and the role of AI is yet to be explored in this context.

The health professions literature contains increasing concern from educators about the potential for inappropriate use of AI by learners, especially during assessment [57]. It is striking that all but one of the psychiatric training organizations contacted for this review did not have a policy for the use of AI in training assessment. Moreover, most psychiatric training organizations also did not have formal guidelines around the use of AI clinically. This will no doubt change in the future but reflects a concerning lag in the safeguards for AI use in psychiatric education. At the time of writing, the HKAM’s policy document remains the gold standard [45]. This wider theme of academic integrity was also largely absent from the included peer-reviewed literature. Rather, several of these records instead emphasized the concern that students might lack the clinical experience and critical thinking skills that can help inoculate against the pitfalls of AI’s biases, inaccuracies, or reductionism.

Finally, the strongest theme throughout the included literature was the need for increased focus in psychiatry curricula on the fundamentals of AI. There is a pressing need for psychiatrists to have strong AI literacy and a familiarity with the AI technology that many of their patients will already be accessing. Developments in clinical psychiatry driven by new AI capabilities, as cited earlier [6,7], have prompted psychiatric educators to reflect on the need to prepare future psychiatrists for a potentially very different clinical setting. The needs of training curricula must be guided by what is occurring at the bedside or in the consulting room.

Despite identification of the need for increased AI content in psychiatric curricula, the implementation of this is unlikely to be straightforward. Curricula are highly complex and interconnected entities, typically requiring substantial design and planning over a prolonged period [58]. A clear challenge will be determining how to embed AI topics into a curriculum in a way that will withstand the ongoing and, at times, very rapid evolution of the field. Furthermore, it is likely that more advanced future versions of AI, such as the development of artificial general intelligence, will necessitate even more important and, perhaps, unpredictable adaptations from educators [59]. The findings included in this scoping review indicate that a focus on translatable basics may be the way forward for psychiatric educators, ensuring foundational health informatics or AI literacy for learners in psychiatry and emphasizing development of critical thinkers who will be able to interpret and appraise any content generated by AI.

### Strengths and Limitations

At the time this study was conducted, the authors were not aware of any published reviews depicting the status quo of the full breadth of learning-based AI in psychiatric education in 2024. This scoping review represents an important snapshot of trends during the early years of scholarly interest in this topic. While additional literature may have been published since the completion of our literature search, the intention of this study was to provide a useful reference point from a specific moment in time to which subsequent work in the field can be compared and contrasted. This also represents a necessary limitation of this study, as it seeks to cover a rapidly evolving field with constant updates of information and swift obsolescence of research findings. Given the field will continue to evolve, it is recommended that updated reviews of the literature be performed at regular intervals to ensure that novel ideas and innovations continue to be identified and explored. Once the field matures beyond primarily commentaries and perspective papers, more systematic approaches to synthesizing the empirical literature may become possible.

### Conclusions

The current evidence base on AI in psychiatric education and training remains at a formative stage. Nevertheless, it is clear that AI will increasingly impact assessment, clinical skills training, and the development of teaching resources in psychiatry. Future curricula in psychiatry will need to incorporate this new knowledge, while educators will need to be cognizant of academic integrity risks, the importance of critical thinking skills, and the necessity to upskill in AI pedagogy. Although the literature considered in this review was cautiously optimistic regarding the role for AI in teaching psychiatry, the knowledge, skills, abilities, and attitudes of psychiatrists and trainees remain underexplored and present a fertile opportunity for future research.

## Supplementary material

10.2196/81517 Multimedia Appendix 1 Further details of literature search strategy.

10.2196/81517 Multimedia Appendix 2 Summary table of data extracted from included records.

10.2196/81517 Checklist 1 PRISMA-ScR (Preferred Reporting Items for Systematic reviews and Meta-Analyses extension for Scoping Reviews) checklist.

## Abbreviations

AI: artificial intelligence
HKAM: Hong Kong Academy of Medicine
LLM: large language model
MCQ: multiple choice question
ML: machine learning
NLP: natural language processing
PAGER: Patterns, Advances, Gaps, Evidence for practice and Research recommendations
PRISMA: Preferred Reporting Items for Systematic Reviews and Meta-Analyses
SCT: script concordance test
VP: virtual patient
