# Supplementary material for: AI in Psychiatric Education and Training From 2016 to 2024: Scoping Review of Trends
Source: JMIR Med Educ. 2025 Dec 31;11:e81517. doi: 10.2196/81517 (PMC12755346; doi:10.2196/81517)
Supplement: Multimedia Appendix 1 [file mededu-v11-e81517-s001.docx]

**Multimedia Appendix 1. Further details of literature search strategy.**

Table S1. Search syntax employed for each database.

| **Database** | **Keyword** | **Syntax Used^a^** |
| --- | --- | --- |
| **PubMed** | Artificial Intelligence | "artificial intelligence"[mh] OR artificial intelligen*[tiab] OR artificially intelligent[tiab] OR AI[tiab] OR large language model*[tiab] OR LLM[tiab] OR machine learning[tiab] OR deep learning[tiab] OR ChatGPT[tiab] OR chatbot*[tiab] |
|  | Psychiatry | "psychiatry"[mh] OR psychiatry[tiab] OR psychiatrist*[tiab] OR psychiatric medicine[tiab] |
|  | Training | "education, medical"[mh] OR training[tiab] OR education*[tiab] OR teaching[tiab] OR learning[tiab] OR assessment[tiab] |
| **Embase** | Artificial Intelligence | (Exp artificial intelligence OR (artificial* intelligen* OR AI OR large language model* OR LLM OR machine learning OR deep learning OR ChatGPT OR chatbot*).ti,ab,kf) |
|  | Psychiatry | (Exp psychiatry OR (psychiatry OR psychiatrist* OR psychiatric medicine).ti,ab,kf) |
|  | Training | (Exp medical education OR (training OR education* OR teaching OR learning OR assessment).ti,ab,kf) |
| **PsycINFO** | Artificial Intelligence | (Exp artificial intelligence OR (artificial* intelligen* OR AI OR large language model* OR LLM OR machine learning OR deep learning OR ChatGPT OR chatbot*).ti,ab,id) |
|  | Psychiatry | (Exp psychiatry OR (psychiatry OR psychiatrist* OR psychiatric medicine).ti,ab,id) |
|  | Training | (medical education.sh OR psychiatric training.sh OR (training OR education* OR teaching OR learning OR assessment).ti,ab,id) |
| **Scopus** | Artificial Intelligence | ("artificial* intelligen*" OR AI OR "large language model*" OR LLM OR "machine learning" OR "deep learning" OR ChatGPT OR chatbot*) |
|  | Psychiatry | (psychiatry OR psychiatrist* OR "psychiatric medicine") |
|  | Training | (training OR education* OR teaching OR learning OR assessment) |

^a^The operator ‘AND’ was used to combine the three listed sub-components of each search.

Table S2. Search syntax employed for Internet search using Google search engine.

| **Syntax Used** | **Limits** |
| --- | --- |
| "artificial intelligence" AND "psychiatry" AND "education" OR "training" | First 20 pages of results scrutinised (due to diminishing relevance of results) |

Table S3. List of specialist psychiatry training organizations contacted.

| **Jurisdiction** | **Name of Organization** |
| --- | --- |
| Australia and New Zealand | Royal Australian and New Zealand College of Psychiatrists |
| Canada | Royal College of Physicians and Surgeons of Canada |
| Hong Kong | Hong Kong College of Psychiatrists, Hong Kong Academy of Medicine |
| India | Indian Psychiatric Society |
| The Philippines | Philippine Psychiatric Association |
| Singapore | College of Psychiatrists, Academy of Medicine Singapore |
| South Africa | College of Psychiatrists of South Africa |
| United Kingdom | Royal College of Psychiatrists |
| United States of America | American Psychiatric Association |
