# Supplementary material for: AI in Psychiatric Education and Training From 2016 to 2024: Scoping Review of Trends
Source: JMIR Med Educ. 2025 Dec 31;11:e81517. doi: 10.2196/81517 (PMC12755346; doi:10.2196/81517)
Supplement: Multimedia Appendix 2 [file mededu-v11-e81517-s002.docx]

**Multimedia Appendix 2. Summary table of data extracted from included records.**

| **Author** | **Year** | **Country** | **Journal/Book/Other** | **Population** | **AI Application** | **Methodology** | **Key Findings** | **Suggestions for Further Research** |
| --- | --- | --- | --- | --- | --- | --- | --- | --- |
| Amos et al [31] | 2024 | Australia | BMC Medical Education | Both under-graduate and post-graduate psychiatry curricula | Using *k*-means clustering to summarise knowledge from successive editions of a core psychiatric textbook into self-organising maps to guide curriculum development | Quantitative | - Educators may resist using machine learning models if they do not understand how they work. - Machine learning techniques are able to distil and visually represent semantic themes from a large knowledge corpus. - Extracting themes from peer-reviewed literature using machine learning may avoid biases inherent in human-selected curricula. | - Incorporating a temporal dimension to the self-organising maps to account for evolving psychiatric nosology. - Formally measuring the entropy in the self-organising maps to assess coherence of the data. |
| Barbe et al [36] | 2023 | Germany | Criminal Justice and Behaviour | Forensic psychiatry trainees (as well as other forensic clinicians) | AI-enhanced VR^a^ training environment for clinical skills | Technical overview of open-source VR framework | - Described technical aspects of a VR framework that combines speech-to-text technology with a natural language processing engine (ChatScript) and a 3D^b^ virtual environment. - This allows a trainee to interact with a simulated patient in a prescribed clinical scenario. | - Establishing tolerability of VR training for participants. - Evaluating perception of verisimilarity. - Adaptation of open-source code by other authors to build on current framework. |
| D’Souza et al [40] | 2023 | Australia, Canada, India | Asian Journal of Psychiatry | Not specified | ChatGPT-3.5^c^ performance on psychiatry clinical case vignettes | Quantitative | - 92% of AI responses were deemed either ‘highly acceptable’ (61%) or ‘moderately acceptable’ (31%). - No AI responses were deemed ‘not acceptable’. - Majority of questions related to management and diagnosis. | - Further research required to build evidence for accuracy of ChatGPT-3.5’s responses to clinical questions. - It is currently uncertain how ChatGPT will respond to prompts relating to atypical populations and/or disorders. |
| Dupuy et al [38] | 2020 | France, Canada | Journal of Affective Disorders | Medical students | Use of Affectiva to analyse students’ facial expressions to assess non-verbal empathy during a virtual psychiatric interview task | Quantitative | - Students displayed significantly more facial emotion when answering questions about the patient compared to questioning or listening to the patient. - AI may be able to provide objective measures of non-verbal empathy during clinical encounters in real time. | - Improvements to the AI algorithm to better distinguish concentration from disgust on facial expressions. - Validation against other measures of empathy. - Longitudinal assessment of effects on clinical skill development. |
| Espejo [32] | 2023 | USA | Academic Psychiatry | Not specified | AI as adjunct tool for education (non-specific) | Opinion (editorial) | - AI can augment small group teaching through generating scenarios, simulating patient responses in real time, or providing information for students to evaluate. - AI may be useful to find answers to learning issues. | - Concerns identified that use of AI has potential to hinder student learning and pose issues for academic integrity. - AI requires monitoring to detect biases. |
| Foster et al [37] | 2016 | USA | Simulation in Healthcare | Medical students | Evaluation of a Natural Language Processing-enhanced virtual patient with depression to assess degree of empathy in medical students | Quantitative | - An AI-enhanced virtual patient can be used to assess empathy in medical students. - Receiving feedback on the degree of empathic responses to a virtual patient led to significantly higher empathy scores in a subsequent human standardised patient encounter (p=0.0277). - Standardised patients rated the students who received feedback more highly than those who did not on empathic statements (p=0.0001), appearing warm and caring (p=0.015), and forming rapport (p=0.004). | - Training of a natural language classifier could enable AI to rate empathic responses instead of relying on human assessors. |
| Gauld et al [20] | 2021 | France, Canada, USA | Psychological Medicine | Not specified | Pedagogical challenges arising from use of AI in psychiatry | Opinion (reply letter) | - Doctors of the future will need to be specifically taught principles for the application and interpretation of AI. - Epistemological basis of AI-based knowledge needs to be clear (concept of explainability). | - Recommendation to develop safeguards around explainability through increased understanding of intelligibility and accountability of AI. |
| Gratzer and Goldbloom [21] | 2020 | Canada | Academic Psychiatry | Psychiatry specialist training | Use of chatbots to deliver therapy, and implications for psychiatry training | Opinion (perspective article) | - Residents should be familiar with the available chatbot therapy options (have a ‘test-drive’). - Residents must be prepared to answer patient questions about chatbot therapy, prescribe a suitable option, and help guide patient use. - Residents must be aware of ethical considerations (e.g. patient privacy) | - One challenge will be establishing a solid evidence-base for specific AI therapies, as individual chatbots may be frequently updated (so literature can quickly become out of date). |
| Herrmann-Werner et al [41] | 2024 | Germany, Oman | Journal of Medical Internet Research | Medical students | Rating ChatGPT-4’s performance on MCQs^d^ using a modified Bloom’s taxonomy | Mixed methods | - ChatGPT-4 correctly answered 92.5% of questions with a detailed stem and 90.6% of those with a short stem. - Questions answered incorrectly were more difficult than those answered correctly. - ‘Remember’ and ‘understand’ were the two levels of the modified Bloom’s taxonomy where ChatGPT-4 made most errors. | - Evaluating how the performance of other LLMs^e^ would compare to ChatGPT-4. - Re-evaluating ChatGPT-4 performance at a later timepoint to determine if the model has improved. |
| Hong Kong Academy of Medicine [45] | 2023 | Hong Kong | Policy statement | Specialist trainee doctors (including psychiatrists) | Policy on the use of LLMs in written assignments | Policy document | - Trainees are responsible for ensuring the originality of their own work. - Use of AI must be acknowledged. - Any AI output must be critically appraised and its accuracy verified by the trainee. | - Assessing bodies should develop alternative assessment modalities that do not rely on written work (such as oral examinations). |
| Hudon et al [44] | 2024 | Canada | JMIR Medical Education | Medical students | Comparison of SCTs^f^ designed by ChatGPT-3.5 and clinical experts | Mixed methods | - SCTs generated by ChatGPT-3.5 and clinical experts were rated as equivalent (for both scenario and question components). - ChatGPT-3.5 and clinical experts also received similar qualitative feedback on strengths and weaknesses (e.g., good fidelity to diagnostic criteria, but questions simplistic). | - Need to explore acceptability of using generative AI in SCT creation. - Use of different prompts or LLM may yield different results. |
| Khanna et al [22] | 2021 | Australia,  USA | Convergence Mental Health (Book) | Psychiatrists of the future | Predictions of how psychiatrists’ workflows will evolve given rapid technological changes and implications for psychiatry training | Opinion (book chapter) | - Psychiatry trainees have limited exposure to clinical informatics (including AI). - Future psychiatrists will need training in how AI can leverage complex data for diagnosis or evaluating treatments (without expectation of achieving expert level knowledge). | - Only USA has a clinical informatics training program currently; other jurisdictions should consider developing similar programs or embedding designated clinical informatics experiences within psychiatry training. |
| Kim et al [23] | 2019 | USA | Academic Psychiatry | Psychiatry specialist training | Perspectives on how educators can prepare trainee psychiatrists for impact on AI on clinical practice | Opinion (perspective article) | - AI likely to be a useful adjunct to clinicians, rather than replace them; training must reflect this. - Need to balance and integrate human values with technology. - Training curricula should be updated to reflect changing practice. | - Experimentation with AI tools should be encouraged. |
| Lemon [30] | 2024 | Australia | International Journal of Social Psychiatry | Not stated | Reflections on how chatbots will influence education in social psychiatry | Opinion (editorial) | - Educational applications of chatbots include rapid generation of clinical scenarios for learning or assessment, and creating a summary of literature for students. - Risks include potential for bias, favouring specific perspectives over others (i.e., biological over social), and inaccuracy of information. - Chatbots complement educators, rather than replace them (educators should emphasise critical thinking skills). | - Examining how chatbots may influence students’ understanding of learning or their clinical judgment. |
| Li et al [42] | 2024 | Taiwan | Psychiatry and Clinical Neurosciences | Psychiatry specialist training | Comparing performance of ChatGPT-4, Bard, and Llama-2 on the Taiwan Psychiatric Licensing Exam and advanced clinical scenario questions | Quantitative | - ChatGPT-4 achieved a passing grade on 2022 exam (69%), but Bard (36%) and Llama-2 (25%) did not. - Bard’s performance was impacted due to refusal to answer 16 questions on specific topics (e.g., suicide and domestic violence). - On advanced clinical scenario questions, psychiatrists would outperform ChatGPT-4 with a 99.2% probability (using Bayesian analysis). | - Pre-training LLMs with psychiatric parameters is likely to increase performance. - Choice of language version of LLM (e.g., Mandarin vs English) has potential to impact results, as does version used and nature of prompt. |
| Louie et al [24] | 2017 | USA | Academic Psychiatry | Psychiatry specialist training | Perspectives on how machine learning technology will change psychiatric practice and, by extension, the training of psychiatrists | Opinion (editorial) | - Machine learning may automate aspects of diagnosis, behavioural observation, and clinical decision-making; training will need to adapt to reflect this. - Trainees must be able to evaluate these technologies and know how to appropriately incorporate them into clinical practice. - Machine learning must become part of the psychiatric curriculum. | - Privacy, patient autonomy, and acceptability concerns will need further examination. - Impact of machine learning technologies on the therapeutic relationship are unknown. - Unclear line of professional responsibility for (i) monitoring data generated by wearables and (ii) being aware of specific features of various AI tools. |
| Luykx et al [43] | 2023 | The Netherlands | World Psychiatry | Not stated | Assessing performance of ChatGPT (unspecified version) on short-answer questions | Quantitative | - ChatGPT answers were rated highly for accuracy (8.4/10), completeness (7.6/10), and nuance (8.1/10). - Psychiatrists/residents using ChatGPT to answer questions scored significantly better than those using non-AI sources (p=0.0016). | - Determining response speed at answering questions when using ChatGPT compared to other resources (ChatGPT hypothesised to be more rapid). |
| Reznick et al [25] | 2020 | Canada | Royal College of Physicians and Surgeons of Canada (RC) | Canadian specialist physicians (including psychiatrists) and trainees | Task force report on AI and digital technologies for Canadian specialist physicians and trainees | Mixed methods | - Technological advancements from AI will necessitate re-training in new specialties for many medical practitioners. - 22.5% of RC Fellows considered that AI education should be a priority topic in residency. - ‘Technical training related to your specialty’ was the highest ranked AI education topic by RC Fellows and residents. | - Medical practitioner competencies (eg CanMEDs) will need to be updated to take into consideration AI. |
| Schildkrout [39] | 2024 | USA | The Journal of Nervous and Mental Disease | Psychiatry specialist training | Potential application for AI-based deidentification technology to enhance clinical psychiatry education | Opinion (clinical perspective) | - Emerging AI technology may allow for recorded patient encounters to be deidentified through facial alteration, but still retain relevant mental state features for educational value. - This could result in a video encyclopaedia of behavioural syndromes and mental state features. | - Emerging technologies in other fields will need to be repurposed to psychiatry. - Compliance with regulatory frameworks will need to be assessed. - Consent processes will need updating. |
| Smith et al [33] | 2023 | Switzerland, UK | International Journal of Social Psychiatry | Education in social psychiatry | Requesting ChatGPT-3.5 to generate teaching ideas for social psychiatry | Pilot test of LLM capabilities | - ChatGPT may be a quick and effective resource for generating teaching material, including clinical vignettes. - Chatbots may be useful in providing knowledge, enabling group discussions, facilitating self-guided learning, and creating teaching content. | - ChatGPT could be used to synthesise large or complex texts into synopses. - Empirical investigations of chatbots in the psychiatry education setting are needed. - The perspectives of students and teachers should be assessed qualitatively. |
| Starke et al [26] | 2021 | Switzerland, Germany | Psychological Medicine | Not specified | Perspective that teaching history of psychiatry is essential to counter ethical, clinical, and conceptual pitfalls of ML^g^ | Reply letter (to Gauld et al, 2021) | - Understanding the historical context of psychiatry (e.g., development of diagnostic concepts or historical abuses) enhances critical evaluation of ML outputs. - Psychiatrists need sufficient training in computer science and computational psychiatry to be familiar with ML concepts. | - Any use of ML to redefine nosology must reflect on historical context, rather than replicating top-down approaches. |
| Torales and O’Higgins [34] | 2024 | Paraguay | International Journal of Social Psychiatry | Not specified | Reflections on applications and limitations of AI in social psychiatry education, especially in South American context | Reply letter (to Smith et al, 2023) | - ChatGPT may help develop psychiatry teaching resources in languages other than English. - However, this may disadvantage primarily spoken indigenous languages. - Human oversight remains important for interpretation of AI outputs. | - Rise of AI may require a shift to more orally based tasks, rather than written, to maintain academic integrity. |
| Torous et al [27] | 2018 | USA | Academic Psychiatry | Psychiatry trainees | Proposal for the development of a curriculum for teaching clinical informatics (including AI) to psychiatry trainees | Opinion (perspective article) | - Ensuring trainees understand the fundamentals of AI equips them to evaluate and properly use AI clinically. - A curriculum is necessary to achieve this. - A proposed structure includes domains of (i) patient care; (ii) communication; (iii) education; and (iv) practice management. | - Development of a psychiatry informatics curriculum will require input from trainees, educators, hospital leadership, university academics, and others. - Further work on specific competencies, training programmes, and evaluation will be required. |
| Vasilchenko and Chumakov [35] | 2024 | Israel, Russia | International Journal of Social Psychiatry | Not specified | Reflections on applications and limitations of AI in social psychiatry education. | Reply letter (to Smith et al, 2023) | - ChatGPT may be less adept in highly specialised fields, such as social psychiatry. - Human connection and interacting with real patients are essential for developing communication skills. | - Guidelines should be developed for ethical use of AI in medical education. - ChatGPT may need to be trained in sub-specialty data to enhance quality of output. |
| Ventriglio and Ricci [28] | 2024 | Italy | International Journal of Social Psychiatry | Not specified | Reflections on applications and limitations of AI in social psychiatry education. | Opinion (editorial) | - Training on use of AI should become routine part of medical curricula. - Current AI may not grasp the full complexity of a humanistic and holistic biopsychosocial framework. | - AI should be used to develop realistic simulation of clinical cases. |
| Zhang et al [29] | 2023 | Canada | JMIR Formative Research | Academic psychiatrists (as well as other mental health professionals) | Interviewing MHPs^h^ to understand attitudes to AI and to inform educational curricula | Qualitative | - Education of MHPs can reduce fears around the use of AI and increase its uptake. - MHPs require basic knowledge of the development, evaluation, and outcomes of clinical AI tools. - MHPs want to understand the broad applications for AI in clinical care, and value ongoing mentoring in the use of AI after being trained. | - A competency-based AI curriculum should be developed. - Further qualitative data should be sought from other sites and participants with different demographics to determine generalisability. |

^a^VR: virtual reality.

^b^3D: three-dimensional.

^c^GPT: generative pre-trained transformer.

^d^MCQs: multiple choice questions.

^e^LLMs: large language models.

^f^SCTs: script concordance tests.

^g^ML: machine learning.

^h^MHPs: mental health professionals.
